# A Drug–Drug Multicomponent Crystal of Metformin and Dobesilate: Crystal Structure Analysis and Hygroscopicity Property

**DOI:** 10.3390/molecules27113472

**Published:** 2022-05-27

**Authors:** Lan Jiang, Xiangnan Hu, Linhong Cai

**Affiliations:** 1 College of Environment and Resources, Chongqing Technology and Business University, Chongqing 400067, China; jianglan@ctbu.edu.cn; 2 Department of Medicinal Chemistry, School of Pharmacy, Chongqing Medical University, Chongqing 400016, China; huxiangnan@cqmu.edu.cn

**Keywords:** drug–drug multicomponent crystal, metformin, dobesilate, diabetic retinopathy, hydrogen-bonding interactions, hygroscopicity property

## Abstract

A drug–drug multicomponent crystal consisting of metformin (MET) and dobesilate (DBS) was prospectively connected by solvent cooling and evaporating co-crystallization using the multicomponent crystal strategy, not only to optimize the physicochemical properties of single drugs, but also to play a role in the cooperative effect of DBS with the potential vascular protective effects of MET against diabetic retinopathy (DR). The crystal structure analysis demonstrated that MET and DBS were coupled in a 3D supramolecular structure connected by hydrogen-bonding interactions with a molar ratio of 1:1. Almost all hydrogen bond donors and receptors of MET and DBS participated in the bonding, which hindered the combination of remaining potential hydrogen bond sites and water molecules, resulting in a lower hygroscopicity property than MET alone.

## 1. Introduction

The latest tenth edition of the Diabetes Atlas of the International Diabetes Federation showed, that in 2021, the global diabetes adult patients reached 537 million; every 5 s, one person died of diabetes or related complications [1]. The negligence of the threat posed by diabetes and its complications is noteworthy. The increase in the prevalence of many diseases is related to the occurrence of diabetes, including cardiovascular disorder, diabetic neuropathy (DNP), retinopathy (DR), kidney disease (DKD), and so on [2,3]. This underlines the importance of decreasing the risk of complications meaningfully, while controlling blood glucose in the development of candidate pharmacological treatments for type 2 diabetes mellitus, which would be a major contribution to the increased prevalence of diabetes [4].

Diabetic retinopathy (DR), a leading cause of irreversible blindness in working-aged people, is an exclusive microvascular complication of diabetes [5]. Epidemiologic studies show the prevalence of DR in diabetic patients, which reaches 34.6%, of which the proportion of severe vision-threatening stages of DR is 10.2% [6]; however, intensive glycemic control alone cannot completely prevent the occurrence and delay the progression of DR in practice [7]. The high morbidity and incidences of this condition bring a tremendous socioeconomic burden to healthcare and society [8].

**Figure 1:**
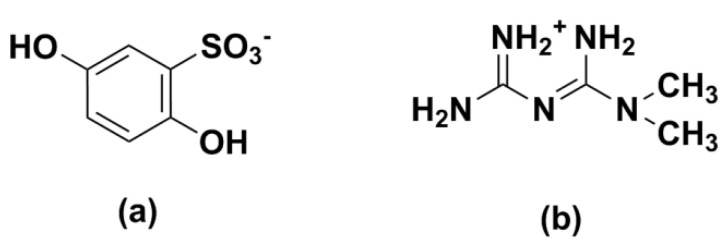
The chemical structures of DBS (**a**); and MET (**b**) with basic and acidic sites highlighted, respectively.

The golden time against the microcirculatory dysfunction is to ameliorate diabetic microcirculation as early as possible in the process of disease, and many trials approve the calcium salt, Dobesilate (DBS, Figure 1a), as a positive way to defeat the early stages of DR [9,10]. It is involved in the treatment of various pathogenic pathways of DR, showing the remarkable advantages of diversified pathways activated by hyperglycemia which treat the disease simultaneously [11].

Metformin (MET, Figure 1b), the most widely prescribed antihyperglycemic agent, is recommended by guidelines of many countries and international organizations as a first-line monotherapy for type 2 diabetes mellitus worldwide [12]. The research shows that it is beneficial in the reduction of severe stages of DR with long-term oral administration [13], which may be attributed to the inducing of alternative splicing of the vascular endothelial cell growth factor A (VEGF-A) [14].

Except for the improvement of visual acuity and fundus symptoms, the reduced incidence of severity progression of DR, such as macular edema, is reflected in clinical research of treatment metformin with calcium dobesilate [15], which is beneficial to prove the rationality of the combination of the two single drugs.

The drug–drug multicomponent crystal is a definition of multicomponent crystals, particularly multicomponent crystals containing combined drugs, which show great potential in pharmaceutical preparations [16,17], and can not only optimize the physicochemical properties of single-component drugs, but also benefit from the pharmacological effects compared with compound preparations, giving credence to its giant advantages over single drug or non-drug multicomponent crystals [18,19].

Good properties may result from the establishment of intermolecular interactions between components, the drug–drug multicomponent crystal that is different from single drug crystals carries out molecular recognition and assembly through noncovalent bonds including hydrogen bonds, van der Waals forces, pi–pi stacking, and electrostatic forces without destroying covalent bonds [20]. Among these interactions, hydrogen bonds with characteristics of high strength, directivity, saturation, and various bonding modes become the most considerable power in the generation of drug–drug multicomponent crystals [21,22]. An infinite one-dimensional hydrogen bond chain of the drug–drug multicomponent crystal formed by metformin and gliclazide could be observed based on hydrogen-bonding interactions, in which metformin is sandwiched by gliclazide to form a hydrophilic channel structure, which provides evidence for the improvement of the solubility of glimepiride and the decrease of the hygroscopicity of metformin [18]. Not only does this affect the optimization of physicochemical properties, but another drug–drug multicomponent crystal connected by glimepiride and metformin also found that, in addition to the enhancement of insulin secretion, the new drug has a stronger inhibitory effect on breast cancer cells and CAL-148 xenografts than metformin and glimepiride alone, or in combination [19,23].

The drug–drug multicomponent crystal of MET–DBS was prospectively yielded for the first time based on the above two ideas of drug combinations and multicomponent crystal technology. The participation of hydrogen bonds was preliminarily surveyed by Fourier-transform infrared spectroscopy (FT-IR), and ^1^H NMR spectroscopy analysis further clarified the molecular composition. Single crystal X-ray diffraction (SCXRD) systematically defined the internal bonding law and the structural difference between the new compound and previously determined structures of MET and DBS salts, the experimental powder X-ray diffraction (PXRD) matched the simulated pattern from single crystal, and the relationship between hydrogen-bonding interactions and hygroscopicity property was conducted by Hirshfeld surface and Dynamic vapor sorption (DVS).

## 2. Results and Discussion

### 2.1. The Absorption Spectrum Characterization Analysis

**Figure 2:**
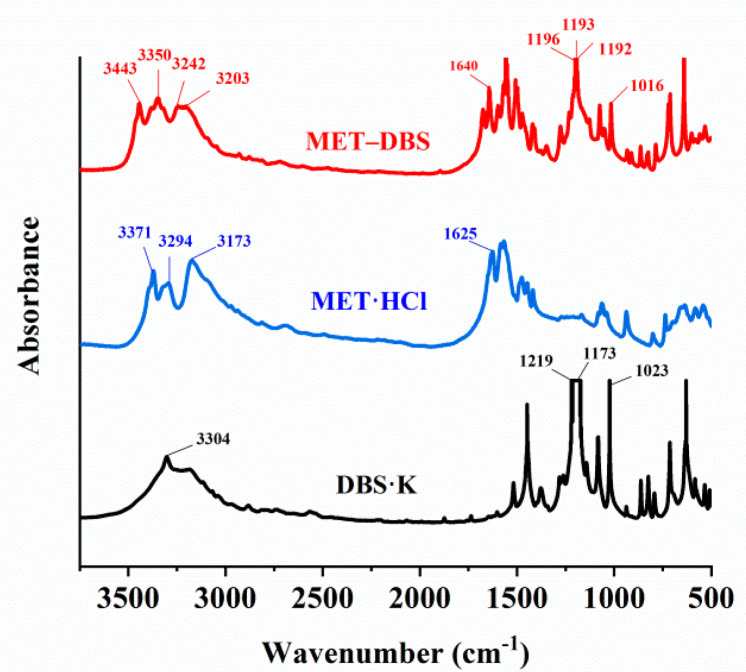
The FT-IR spectrum of DBS·K, MET·HCl, and MET–DBS indicated by black, blue, and red lines, respectively.

According to the characteristic and fingerprint region of the FI-IR spectrum, we identified and distinguished the characteristic functional groups of the drug–drug multicomponent crystal of MET–DBS and two single drugs, and the differences in absorption peaks between them were found and analyzed, indicating the generation of a new compound, as shown in Figure 2.

The frequency range of the C=N stretching vibration (generally 1670–1600 cm^−1^) was shifted from 1625 cm^−1^ in MET·HCl to 1640 cm^−1^ in MET–DBS, which moved slightly to a higher frequency. At the same time, the stretching vibration frequency of N-H in MET·HCl was transferred from 3371, 3294, 3173 cm^−1^ to 3443, 3350, 3203 cm^−1^ in MET–DBS, which may be due to the influence of the charge assisted hydrogen bond in MET·HCl, resulting in its vibration frequency being lower than that in MET–DBS.

There were two characteristic absorption peaks in DBS·K, which were the stretching vibration of the sulfonyl and phenolic hydroxyl groups. The asymmetric stretching vibration absorption of S=O was 1219–1173 cm^−1^ with an intense and broadened peak. The symmetric stretching vibration of S=O was 1023 cm^−1^ with a medium to strong intensity sharp peak. The corresponding wavenumbers of these two vibrations in MET–DBS were 1196–1192 cm^−1^ and 1016 cm^−1^, with frequency and intensity slightly decreased, which may have been caused by the participation of S=O in the hydrogen bonding in MET–DBS. The stretching vibration of O-H changed from 3304 cm^−1^ to 3242 cm^−1^ in the drug–drug multicomponent crystal with a broad medium intensity peak, which may be attributed to the increase in the strength of H-bonds, accompanied by shifts to lower frequencies of the absorption bands.

^1^H NMR spectroscopy analysis was further performed to determine the chemical structure of a new drug–drug compound of MET–DBS compared with the hydrogen composition of MET·HCl and DBS·K.

Compared with the two single drug molecules, there were three chemical shifts in the drug–drug multicomponent crystal of MET–DBS that had not changed, 2.92 (s, 6H), 9.80 (s, 1H), and 8.80 (s, 1H), respectively, representing six hydrogen atoms of methyl groups in MET·HCl, and two hydrogen atoms of hydroxyl groups in DBS·K.

The six hydrogen atoms in the 6.64–6.50 multiple absorption peaks (m, 6H) were attributed to two NH_2_ groups in the guanidine group of MET·HCl, two hydrogen atoms in the benzene ring of DBS·K, and another hydrogen atom in the benzene ring which was located at 6.87 (s, 1H). The other two hydrogen atoms of the amino group with relatively high chemical shifts in MET·HCl were transferred to 7.18 (s, 2H) in the drug–drug multicomponent crystal.

**Figure 3:**
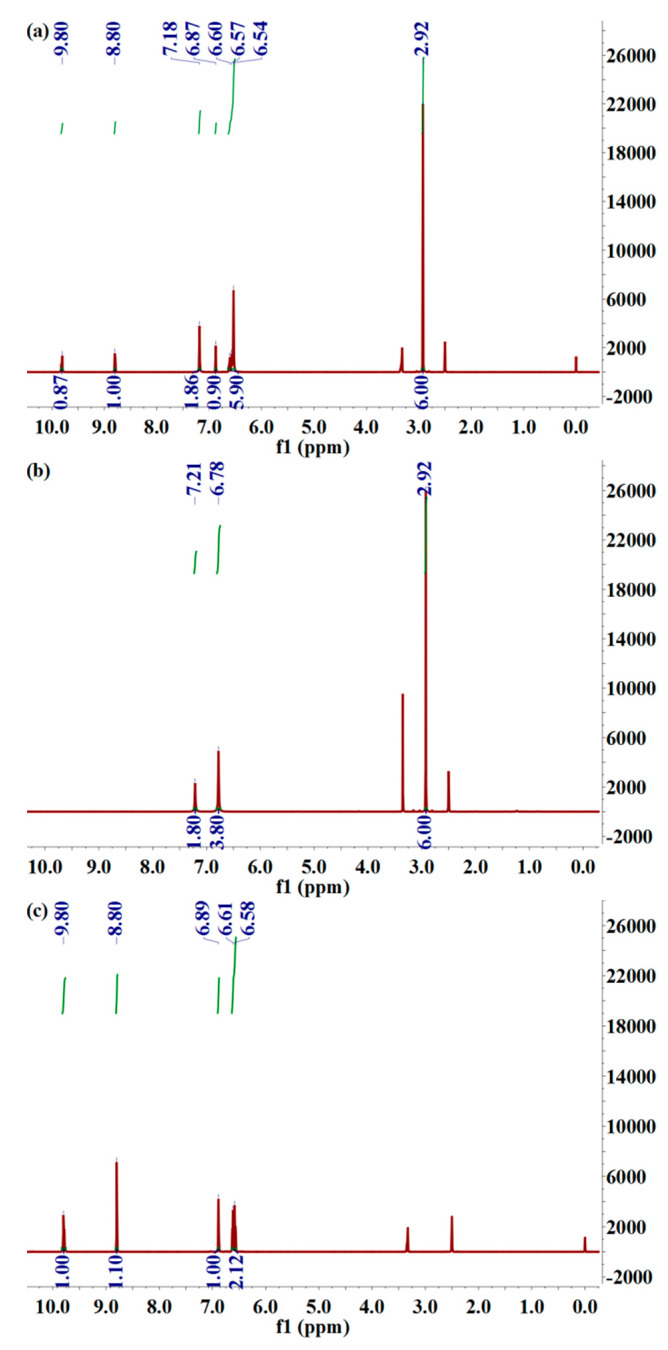
^1^H NMR spectra for drug–drug crystal of (**a**) MET–DBS, (**b**) MET·HCl, and (**c**) DBS·K.

Therefore, they were all hydrogen atoms in the two single drug molecular structures in MET–DBS, the deviation of the chemical shift was not obvious compared with the single drugs, and it was proven that the molar ratio between MET and DBS in the drug–drug multicomponent crystal was 1:1, as shown in Figure 3. For detailed NMR data, see ^1^H NMR Spectroscopy Analysis in Materials and Methods.

### 2.2. The Single Crystal Structure Analysis

**Figure 4:**
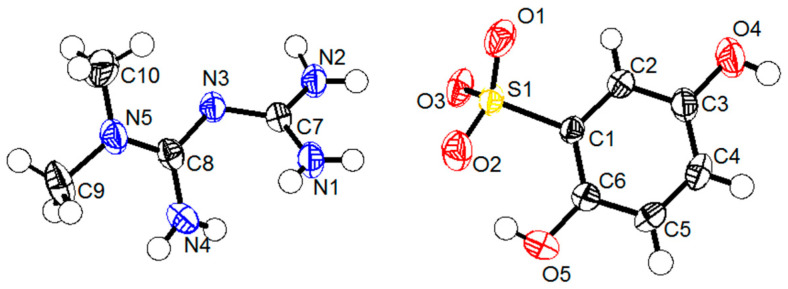
A drug–drug multicomponent crystal of MET–DBS shown at 50% probability ellipsoids.

The salts of MET and DBS were used as crystalline materials, and the anion exchange method was expected to prepare phase-pure MET–DBS drug–drug multicomponent crystals. After cooling and evaporating crystallization, the yellow block-shaped crystals appeared after about 2 weeks, and the crystal size chosen for the diffraction test was 0.17 × 0.12 × 0.1 mm^3^, which was suitable for SCXRD analysis. The detailed crystallographic data and structural refinement were given in Table 1. The asymmetric unit in Figure 4 showed that the drug–drug multicomponent crystal was composed of MET and DBS in a molar ratio of 1:1, which was consistent with the ^1^H NMR spectra. The hydrogen atom in the sulfonic acid group of DBS was transferred to the imino group of MET, which further proved that it was a salt-type multicomponent crystal. The pKa difference between bases and acids, if ΔpKa > 3, is usually used as an indicator of salt formation [24]. The pKa values of MET and DBS are 12.4 [25] and −2.8 [26], respectively, which corresponds to a ΔpKa of 15.2, reasonably indicating that proton transfer occurs between the two components to form the salt.

**Figure 5:**
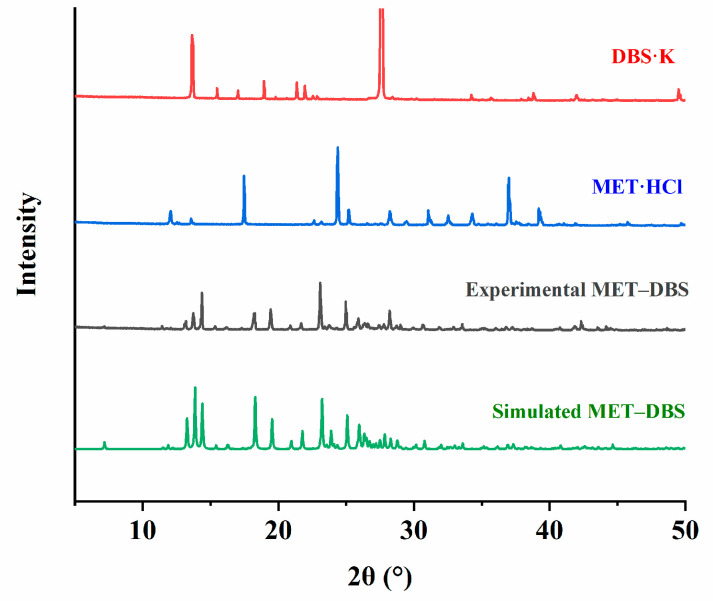
Powder X-ray diffraction patterns of DBS·K, MET·HCl, and MET–DBS (experimental and simulated).

From the RXRD patterns in Figure 5, it was determined that this new compound, characterized as 2θ at 13.73°, 14.37°, 18.27°, 19.44°, 23.1°, and 24.97°, well matched the simulated pattern calculated from the single crystal structure, which was unique from the characteristic peaks of MET·HCl (12.07°, 17.47°, 24.38°, 36.99°, and 39.3°) and DBS·K (13.64°, 18.94°, 21.35°, 21.95°, and 27.63°), showing that the prepared crystals were phase-pure.

As shown in Table 2, the single crystal was mainly composed of eight kinds of hydrogen bond interactions to maintain the crystal structure, including an intramolecular hydrogen bond existing in DBS with an O5·O2 distance of 2.665(3) Å, ∠(O5-H5-O2) = 148.6°; therefore, for DBS, all oxygen atoms in its molecular structure participated in the formation of hydrogen bonds, whereas all nitrogen atoms of MET except for N5 participated in the connection of noncovalent bonds, which fully showed the role of hydrogen bonds in the production of the drug–drug multicomponent crystal, so its one-dimensional, two-dimensional, and three-dimensional hydrogen-bonded arrangement was continued to make a more detailed analysis.

**Figure 6:**
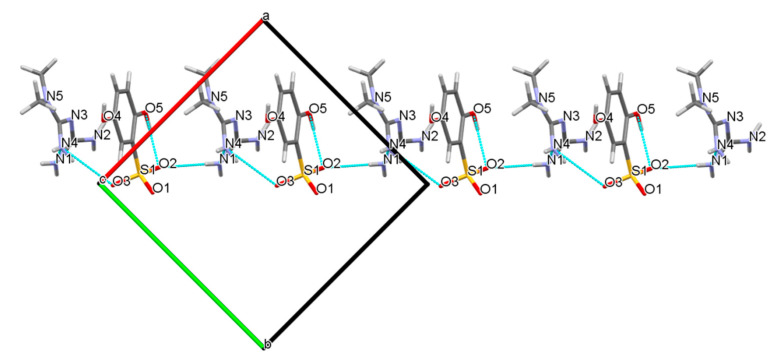
The one-dimensional hydrogen-bonded chain of MET–DBS viewed along the c axis.

In the one-dimensional chain along the c-axis drawn in Figure 6, each MET and one DBS ion were arranged in pairs. The donors N1 and N4 in the two amino groups of MET provided a hydrogen atom, respectively, forming N1-H1A···O2 and N4-H4B···O3 hydrogen bonds with O2 and O3 in the sulfonyl group receptor of DBS with N1···O2 and N4···O3 distances of 3.075(3) and 3.061(3) Å, respectively, and infinitely extended into a primary motif chain structure.

**Figure 7:**
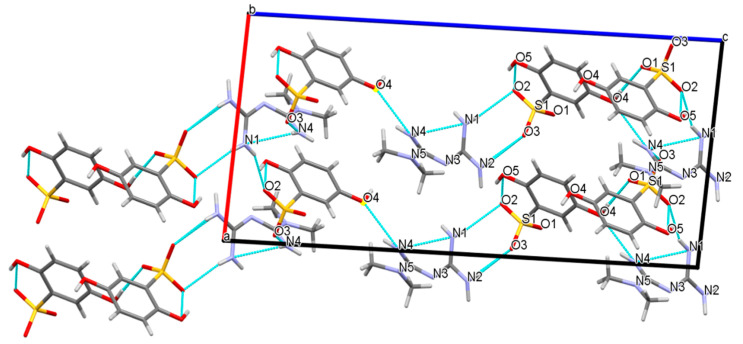
The two-dimensional hydrogen-bonding layered structure composed of MET and DBS along the b axis.

Based on the one-dimensional chain structure, O1 in the sulfonyl group of one DBS anion and N4 in the phenolic hydroxyl group of one MET cation interacted with another DBS anion, respectively, to form O4-H4···O1 and N4-H4A···O4 hydrogen bonds with O4···O1 and N4···O4 angles of 172.3° and 140(3)° in Figure 7. In addition to this chain connection, a dimer described as an $R_{2}^{2}$(8) ring could be formed between MET and DBS ions, which were contributed by the proton transfer from the N1 and O3 atoms, which connected via self-assembly of two strong charge-assisted hydrogen bonds N1-H1B···O2 and N2-H2B···O3, r(N1···O2) = 3.075(3) Å and r(N2···O3) = 2.988(2) Å, respectively, indicating the possibility of a hydrogen-bonded supramolecular synthon formed by a guanidine group and sulfonyl group. The four hydrogen bond interactions described above were used further, mainly to derive and expand into a two-dimensional layer along the a and c axis. To determine the formation ratio, we could also found that in a 2D structure, one DBS anion interacted with up to three MET cations, forming N4-H4B···O3, N1-H1A···O2, and N4-H4A···O4 hydrogen bonding interactions. Similarly, up to three DBS anions were combined with one MET cation, which mainly depended on the four hydrogen bonds of N4-H4B···O3, N1-H1A···O2, N1-H1B···O2, and N2-H2B···O3. DBS itself was only connected with another DBS anion through the O4-H4···O1 hydrogen bond, r(O4···O1) = 2.689(2) Å, but we could see that all hydrogen bond donors and receptors of DBS have formed intermolecular or intramolecular hydrogen bonds with MET cations or DBS itself, which proved that DBS was an ideal and promising crystal co-former.

**Figure 8:**
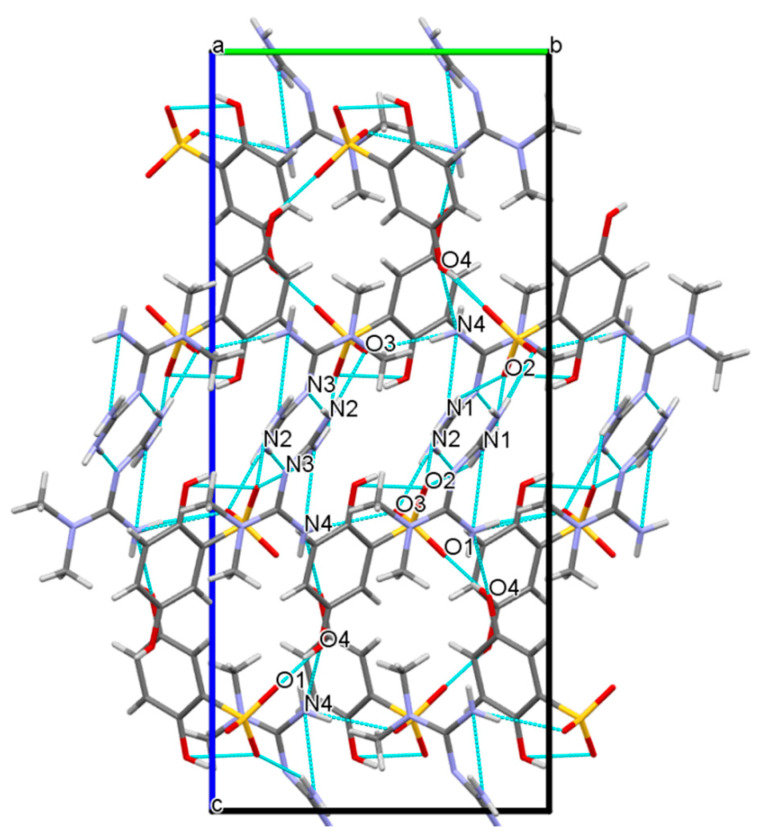
The 3D packing diagram connected by hydrogen bonds of MET–DBS viewed along the a axis.

The two-dimensional surface formed a further three-dimensional stacking architecture structure through the $R_{2}^{2}$(8) ring drawn in Figure 8, which was a centrosymmetric dimer unit equipped by two MET cations through the N2-H2A···N3 hydrogen bond, r(N2···N3) = 3.123(3) Å, ∠(N2-H2A-N3) = 174(2)°. So far, all hydrogen bond donors and receptors in MET, except for the N5 atom, were also involved in the generation of noncovalent bonds, indicating that MET and DBS relied on hydrogen bond interactions to establish a close relationship. Further observing the three-dimensional packing, it was found that in the drug–drug crystal hydrogen bonding arrangement structure, each DBS was connected with two DBS anions by O4-H4···O1 interactions through O4 and O1, respectively. Moreover, each MET or DBS was not connected with three hetero ions at most, but they constructed a complex hydrogen-bonding network with four hetero ions through N4-H4A···O4, N4-H4B···O3, N1-H1A···O2, and the hydrogen bonds forming the dimer between MET and DBS, which also showed that the molar ratio between MET and DBS, was 1:1.

**Figure 9:**
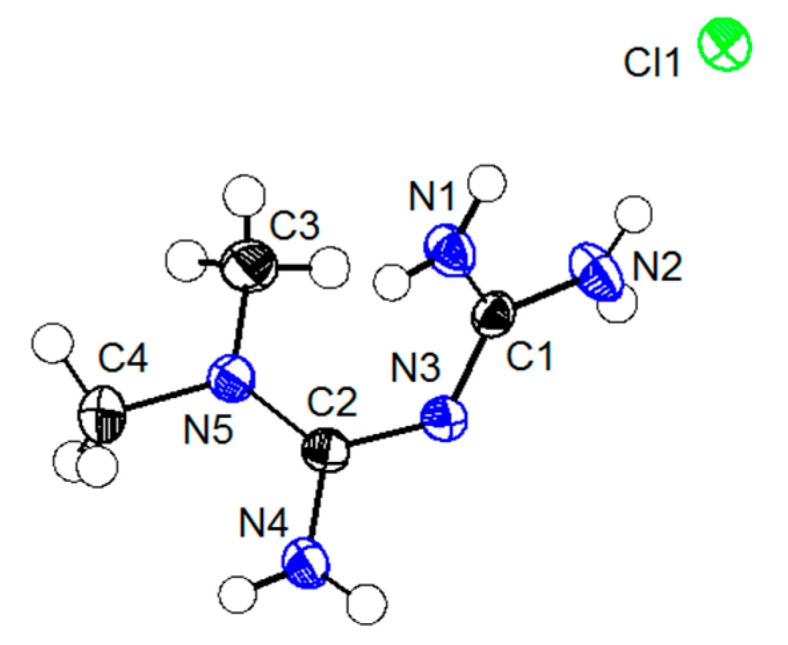
A single crystal of MET·HCl shown at 50% probability ellipsoids.

**Figure 10:**
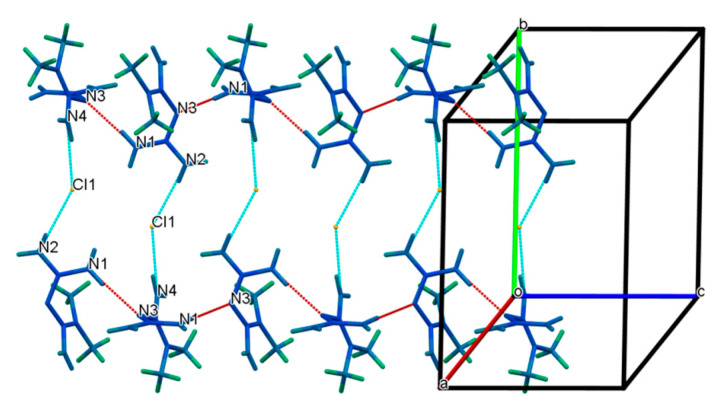
Hydrogen bonded two-dimensional layered structure of MET·HCl.

**Figure 11:**
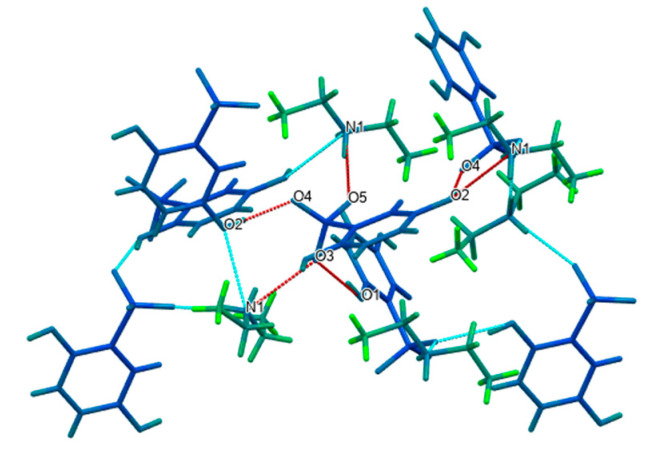
The 3D packing structure of the diethylammonium salt of DBS.

To better compare the differences in the crystal structures of the multicomponent and single drug molecules, the single crystal of MET·HCl (CCDC no. 2170425) was obtained experimentally, and the diethylammonium salt of DBS [27] was found in the Cambridge Crystallography Data Center (CCDC). The dihedral angle of C1-N3-C2-N5 in the asymmetric unit of MET·HCl, shown in Figure 9, was 55.9°. Compared with the ellipsoid structure of MET–DBS in Figure 4, the C7-N3-C8-N5 dihedral angle of MET in MET–DBS was 150.9°. The –C(NH_2_)_2_ groups of MET in the two crystal forms were almost in the opposite direction due to the different dihedral angles, but the single crystal of MET–DBS existed in the same conformation as other drug–drug multicomponent crystals containing MET cations as reported in CCDC [28]. Additionally, MET in the single crystal of MET–DBS was established in a centrosymmetric dimer unit rather than extending the linear arrangement as in the MET·HCl crystal form in Figure 10. In the case of another multicomponent crystal of DBS, Figure 11 showed a 3D packing structure in which each DBS anion formed N-H···O and O-H···O hydrogen bonds with three diethylammonium cations and three DBS anions, respectively. As a result, all hydrogen bond suppliers and receptors in DBS participated in bonding, similarly to MET–DBS, thus further demonstrating the rationality of DBS as an ideal crystal co-former.

### 2.3. The Structure-Property Analysis

**Figure 12:**
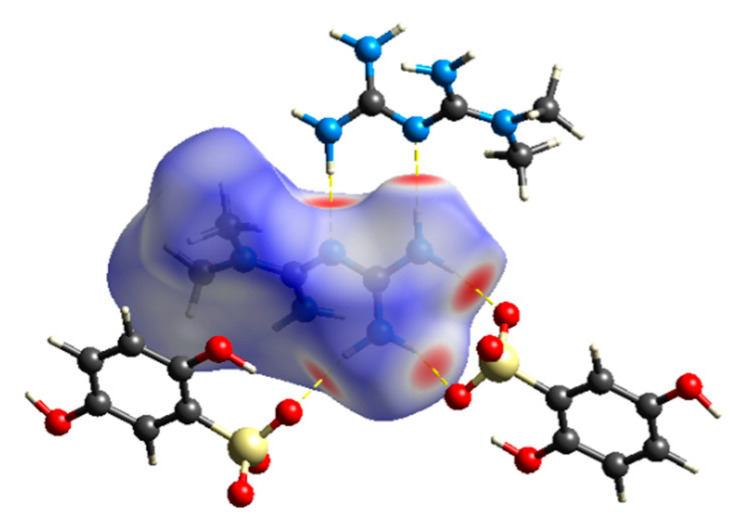
The Hirshfeld surface marked with hydrogen bonding interactions of MET–DBS.

**Figure 13:**
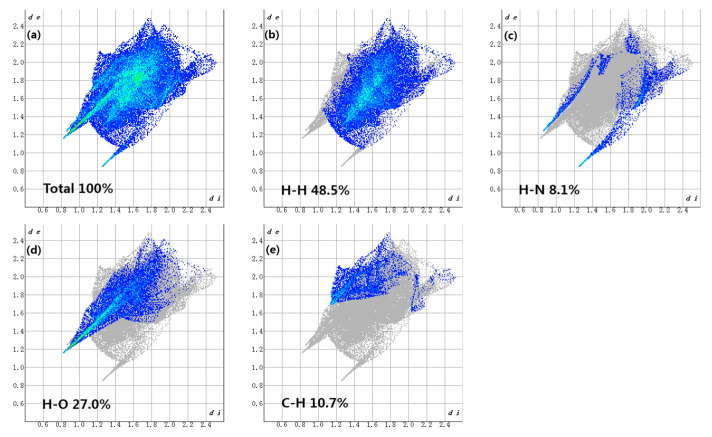
The 2D fingerprint plots of the overall proportion of various hydrogen bond types of MET–DBS highlighted close contacts from H atom to H, N, O, and C atoms.

To further explore the molecular internal environment and intermolecular interaction of the drug–drug multicomponent crystal, both qualitatively and quantitatively, the Hirshfeld surface analysis and calculation of MET–DBS were carried out by using crystal Explorer 17.5 software [29]. Taking the MET in the crystal as the calculation input object, the corresponding Hirshfeld surface map and 2D fingerprint spectrum of the crystal were obtained visually, as shown in Figure 12 and Figure 13.

In the Hirshfeld surface map, the dark red spots signified the strongest intermolecular interactions, and the MET established a relationship with DBS via N-H···O as the main hydrogen bond force, including O-H···O bonds between MET. The relatively weak interactions on the surface, represented by white and blue areas, were gradually weaker than van der Waals forces. By analyzing the unique 2D fingerprint spectrum, we could see that the main intermolecular interactions in the formation of MET–DBS were H···O and H···H interactions. The proportion of H···H, C···H, and H···N in the whole was 48.5%, 10.7%, and 8.1%; H···H accounted for a half of the whole, suggesting that the molecular surface was mainly composed of a series of H atoms.

A large number of H atoms on the molecular surface promoted the contribution of the strong H···O bond, which was nearly 1/3, which made it the main noncovalent driving force for drug–drug supramolecular structure formation, presented as one spike in Figure 13d, indicating that crystal co-formers with a high content of oxygen elements such as carboxyl, sulfonyl, and hydroxyl groups, may be more closely connected with MET to structure drug–drug multicomponent crystals.

**Figure 14:**
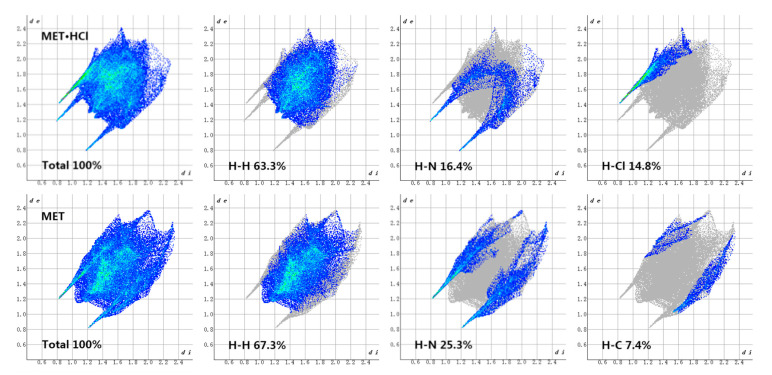
Highlight close contacts from H atom to H, N, Cl, and C atoms in the 2D fingerprint plots of MET in MET·HCl and single component of MET.

The hydrogen-bonding interactions also existed in MET·HCl, and the main hydrogen bond donors and acceptors of MET in the structure of MET·HCl, or MET–DBS, all came from the N atoms, so the related interactions were counted, such as the N···H and Cl···H interactions in MET·HCl, and the N···H and O···H interactions in MET–DBS. In Figure 13, the sum of N···H and O···H interactions in MET–DBS accounted for 35.1% of the total interactions, and as shown in Figure 14, the sum of N···H and Cl···H interactions in MET·HCl accounted for 31.2%; both of the proportions of hydrogen bond interactions that participated in MET were similar. However, the contribution of interactions related to N atoms in the single component crystal structure of MET [30] was only 25.3% in Figure 14, which was a 5.9% and 9.8% difference from that of MET·HCl and MET–DBS, respectively. It has been reported in various studies that MET is a hygroscopic material; at 80% relative humidity (RH), the weight change can reach 60%, resulting in the deliquescence and inactivation of MET [18,23,31]. Compared with MET·HCl and MET–DBS, the proportion differences between interactions may increase the possibility of uncombined hydrogen bond binding sites in the single component crystal of MET potentially binding with water molecules in the air, resulting in higher moisture absorption. To prove the influence of hydrogen-bonding interactions as one of its properties, we carried out the DVS experiment on MET·HCl and MET–DBS.

**Figure 15:**
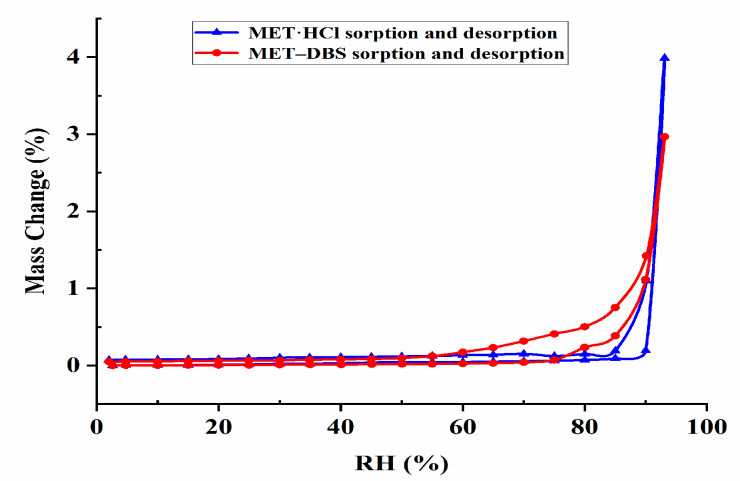
The dynamic vapor adsorption plot for MET·HCl and drug–drug multicomponent crystal of MET–DBS.

DVS was a dynamic analysis used to study the weight change of drugs caused by water absorption with an increase in humidity. In Figure 15, before the RH reached 80%, the weight change of MET·HCl and MET–DBS was no more than 0.5%, showing low moisture absorption. At 90% RH, both compounds absorbed a large amount of water vapor, MET·HCl had a 3.9% weight change, and exhibited a little higher moisture absorption than MET–DBS which had a 2.9% weight change, which may be related to the 3.9% difference in hydrogen-bonding interactions between MET·HCl and MET–DBS in Figure 13 and Figure 14. Moreover, with the role of salt formation, these two compounds all established more abundant and stable hydrogen bond networks than MET alone to close potential hydrogen bonding sites, which overcame the hygroscopic property of MET and stabilized under ambient humidity.

## 3. Materials and Methods

### 3.1. Materials

Potassium 2,5-dihydroxybenzenesulfonate (DBS·K) was purchased from Bide Pharmatech Ltd. (Shanghai, China). Metformin hydrochloride (MET·HCl) was received from Adamas Reagent Company (Shanghai, China). The solvent water was accessed from Milli-Q Advantage A10 Water Purifier and all chemicals were used without further purification.

### 3.2. Methods of Synthesis

MET·HCl (165.62 g/mol, 10 mmol, 1.66 g) and DBS·K (228.26 g/mol, 10 mmol, 2.28 g) were weighed into a 50 mL single mouth eggplant flask, adding 10 mL of pure water, drop by drop, and stirring until dissolved. Under the conditions of an ambient temperature, we continued stirring for 3 h, vacuum filtration occurred to remove insoluble impurities, the filtrate was transferred to a 10 mL round bottom centrifuge tube uncovered and placed in a 4 °C refrigerator undisturbed, cooling and evaporating occurred slowly for crystallization, then yellow block-shaped crystals appeared after about 2 weeks, which were suitable for SCXRD analysis and further characterization studies.

### 3.3. Methods of Absorption Spectrum Characterization Analysis

#### 3.3.1. Fourier-Transform Infrared (FT-IR)

Each 1–2 mg of powder sample was ground with dried KBr powder and pressed into a tablet. The measurements were carried out using a Nicolet FT-IR spectrometer (Nicolet iS10, Waltham, MA, USA) in a scanning range of 4000–500 cm^−^^1^ with 10 accumulative scans under ambient temperature.

#### 3.3.2. ^1^H NMR Spectroscopy Analysis

The ^1^H NMR spectra were measured with a Bruker AVANCE III NMR spectrometer (Bruker, Karlsruhe, Germany) at 600 MHz with DMSO-d6 as the NMR solvent. The ^1^H NMR data for the drug–drug multicomponent crystal of MET–DBS, MET·HCl, and DBS·K respectively was:

^1^H NMR (600 MHz, DMSO-d6) δ 9.80 (s, 1H), 8.80 (s, 1H), 7.18 (s, 2H), 6.87 (s, 1H), 6.64–6.50 (m, 6H), 2.92 (s, 6H).
^1^H NMR (600 MHz, DMSO-d6) δ 7.21 (s, 2H), 6.78 (s, 4H), 2.92 (s, 6H).
^1^H NMR (600 MHz, DMSO-d6) δ 9.80 (s, 1H), 8.80 (s, 1H), 6.89 (s, 1H), 6.60 (d, J = 17.7 Hz, 2H).

### 3.4. Methods of Single Crystal Structure Analysis

#### 3.4.1. Single Crystal X-ray Diffraction (SCXRD)

SCXRD data were measured on a Bruker SMART CCD diffractometer (Bruker, Karlsruhe, Germany) with Cu-Kα radiation (λ = 1.54184 Å) at 293 K. The structure was analyzed by the Olex2 [32] 1.2 program and the SHELXS [33] structure solution program using direct methods, and reflections were merged by SHELXL [34] according to the crystal class for the calculation of statistics and refinement. Empirical absorption correction using spherical harmonics, were implemented in SCALE3 ABSPACK scaling algorithm. All hydrogen atoms were located using the differential Fourier map and refined with isotropic displacement parameters, and non-hydrogen atoms were revised with anisotropic displacement parameters.

#### 3.4.2. Powder X-ray Diffraction (PXRD)

A Bruker D8 advanced X-ray diffractometer (Bruker, Germany) was utilized to collect powder X-ray diffraction data with Cu-Kα radiation (λ = 1.5406 Å). 40 kV and 40 mA were the voltage and current, respectively. At an ambient temperature and a scan speed of 5°/min, data for each sample were captured in the 2θ range of 5–50° (step size 0.01°).

### 3.5. Methods of Structure-Property Analysis

#### 3.5.1. Hirshfeld Surface Analysis

The 3D Hirshfeld surface of molecules and 2D fingerprint plots were calculated and produced by using the program Crystal Explorer 17.5 software. The parameter of d_e_ and d_i_ were defined, respectively: d_e_, the distance from the point of crystal surface to the nearest atomic nucleus outside the surface; d_i_, the distance from the point of crystal surface to the nearest nucleus inside the surface. Each point on the 2D fingerprint plots was formed by the discrete intervals composed of d_i_ and d_e_ (0.01 × 0.01 Å), with colors ranging from blue to green and red.

#### 3.5.2. Dynamic Vapor Sorption (DVS)

The vapor sorption and desorption profiles of the materials were determined at 25 °C using an Aquadyne DVS analyzer (Quantachrome Instruments, Boynton Beach, FL, USA). Each sample was dried under dry nitrogen purge until a consistent weight was received. Then, the samples of MET·HCl (81.46 mg) and MET–DBS (82.81 mg) were investigated simultaneously at each step size of 5% RH, following the sorption and desorption cycle of 2%–95%–2%. The relative humanity (RH) was shifted to the next step if the weight change within 10 min was less than 0.02%, with an 8 hour maximum equilibrium time.

## 4. Conclusions

The drug–drug multicomponent crystal consisting of MET and DBS with 1:1 stoichiometry was the first generated in this paper. There was a complex hydrogen-bonding network between MET and DBS, which was mainly connected by N-H···O bonds, nearly 1/3 of the driving force was contributed by the H···O bond, as visualized by Hirshfeld surface analysis. The hydrogen-bonding interactions were not only formed between MET and DBS in the $R_{2}^{2}$(8) ring linked by charge-assisted hydrogen bonds, but the MET cations formed centrosymmetric dimers with N-H···N bonds, and DBS anions were diffused by hydrogen bond donors O-H, accompanied by the shifts of O-H stretching vibrations to the lower frequency of the absorption bands in the FT-IR spectrum.

Almost all hydrogen bond donors and receptors in MET and DBS were involved in bonding, which significantly reduced the number of sites in contact with water molecules in the air, thus reducing the hygroscopicity of MET. This structural change resulted in a transformation in its property, showing the great potential of drug–drug multicomponent crystals in the pharmaceutical field. It was worthy of in-depth research to contribute to the development of optimal new medications for diabetic complications.

## Figures and Tables

**Table 1:** Crystal data and structural refinement for MET–DBS.

Empirical formula	C_10_H_17_N_5_O_5_S
Formula weight	319.34
Temperature/K	293(2)
Crystal system	monoclinic
Space group	C2/c
a/Å	10.87589(20)
b/Å	10.9043(2)
c/Å	24.6427(4)
α/°	90
β/°	93.8645(16)
γ/°	90
Volume/Å^3^	2915.83(9)
Z	8
ρ_calc_g/cm^3^	1.455
μ/mm^–^^1^	2.268
F(000)	1344.0
Crystal size/mm^3^	0.17 × 0.12 × 0.1
Radiation	CuKα (λ = 1.54184)
2Θ range for data collection/°	7.19 to 141.83
Index ranges	−13 ≤ h ≤ 10, −9 ≤ k ≤ 13, −28 ≤ l ≤ 30
Reflections collected	5408
Independent reflections	2759 [R_int_ = 0.0233, R_sigma_ = 0.0329]
Data/restraints/paraMETers	2759/1/219
Goodness-of-fit on F^2^	1.062
Final R indexes [I ≥ 2σ (I)]	R_1_ = 0.0424, wR_2_ = 0.1181
Final R indexes [all data]	R_1_ = 0.0478, wR_2_ = 0.1250
Largest diff. peak/hole/e Å^–^^3^	0.34/–0.33
CCDC deposit number	2161712

**Table 2:** Hydrogen bonded geometries for MET–DBS.

D-H···A	d(D-H)/Å	d(H-A)/Å	d(D-A)/Å	D-H-A/◦
O4-H4···O1 ^1^	0.82	1.87	2.689(2)	172.3
O5-H5···O2	0.82	1.93	2.665(3)	148.6
N1-H1A···O2 ^2^	0.81(3)	2.32(3)	3.075(3)	156(3)
N1-H1B···O2 ^3^	0.83(3)	2.32(3)	3.085(2)	155(2)
N2-H2A···N3 ^4^	0.89(3)	2.23(3)	3.123(3)	174(2)
N2-H2B···O3 ^3^	0.848(17)	2.149(18)	2.988(2)	170(3)
N4-H4A···O4 ^5^	0.82(3)	2.25(3)	2.929(3)	140(3)
N4-H4B···O3	0.85(3)	2.26(3)	3.061(3)	159(3)

Symmetry codes: (^1^) 3/2 − X, 1/2 + Y, 1/2 − Z; (^2^) −1/2 + X, 1/2 + Y, + Z; (^3^) 1 − X, 1 − Y, 1 − Z; (^4^) 3/2 − X, 3/2 − Y, 1 − Z; (^5^) 1 − X, + Y, 1/2 − Z.

## Data Availability

The crystallographic information file (CIF) of this study is deposited at the Cambridge Crystallography Data Center with deposit number 2161712 and 2170425. This data can be obtained free of charge via http://www.ccdc.cam.ac.uk/conts/retrieving.html (accessed on 10 April 2022).

## Sample Availability

Samples of the compounds are not available from the authors.
